# Effort Oxygen Saturation and Effort Heart Rate to Detect Exacerbations of Chronic Obstructive Pulmonary Disease or Congestive Heart Failure

**DOI:** 10.3390/jcm8010042

**Published:** 2019-01-04

**Authors:** César Gálvez-Barrón, Felipe Villar-Álvarez, Jesús Ribas, Francesc Formiga, David Chivite, Ramón Boixeda, Cristian Iborra, Alejandro Rodríguez-Molinero

**Affiliations:** 1 Clinical Research Unit, Consorci Sanitari del Garraf, Sant Pere de Ribes, PC 08810 Barcelona, Spain; arodriguez@csg.cat; 2 Department of Pulmonology, IIS Fundación Jiménez Díaz, CIBERES, UAM, PC 28040 Madrid, Spain; FVillarA@quironsalud.es; 3 Servei de Pneumologia, Hospital Universitari de Bellvitge, IDIBELL, L’Hospitalet de Llobregat, PC 08907 Barcelona, Spain; jribass@bellvitgehospital.cat; 4 Geriatric Unit. Internal Medicine Department, IDIBELL, Unversitat de Barcelona, Hospital Universitari de Bellvitge, L’Hospitalet de Llobregat, PC 08907 Barcelona, Spain; fformiga@bellvitgehospital.cat (F.F.); dchivite@bellvitgehospital.cat (D.C.); 5 Internal Medicine Department, Hospital de Mataró-Consorci Sanitari del Maresme, PC 08304 Barcelona, Spain; rboixedaviu@gmail.com; 6 Cardiology Department, IIS Fundación Jiménez Díaz, PC 28040 Madrid, Spain; cristian.iborra@salud.madrid.org

**Keywords:** chronic obstructive pulmonary disease, heart failure, diagnostic algorithms

## Abstract

Background: current algorithms for the detection of heart failure (HF) and chronic obstructive pulmonary disease (COPD) exacerbations have poor performance. Methods: this study was designed as a prospective longitudinal trial. Physiological parameters were evaluated at rest and effort (walking) in patients who were in the exacerbation or stable phases of HF or COPD. Parameters with relevant discriminatory power (sensitivity (Sn) or specificity (Sp) ≥ 80%, and Youden index ≥ 0.2) were integrated into diagnostic algorithms. Results: the study included 127 patients (COPD: 56, HF: 54, both: 17). The best algorithm for COPD included: oxygen saturation (SaO_2_) decrease ≥ 2% in minutes 1 to 3 of effort, end-of-effort heart rate (HR) increase ≥ 10 beats/min and walking distance decrease ≥ 35 m (presence of one criterion showed Sn: 0.90 (95%, CI(confidence interval): 0.75–0.97), Sp: 0.89 (95%, CI: 0.72–0.96), and area under the curve (AUC): 0.92 (95%, CI: 0.85–0.995)); and for HF: SaO_2_ decrease ≥ 2% in the mean-of-effort, HR increase ≥ 10 beats/min in the mean-of-effort, and walking distance decrease ≥ 40 m (presence of one criterion showed Sn: 0.85 (95%, CI: 0.69–0.93), Sp: 0.75 (95%, CI: 0.57–0.87) and AUC 0.84 (95%, CI: 0.74–0.94)). Conclusions: effort situations improve the validity of physiological parameters for detection of HF and COPD exacerbation episodes.

## 1. Introduction

HF and COPD are among the chronic conditions with the highest adverse impact on the population [1,2,3]. HF and COPD exacerbations negatively influence survival, autonomy, and quality of life of individuals and are a common cause of hospitalization and visits to the emergency department [4,5,6].

Several detection models based on remote monitoring of clinical parameters such as dyspnea, HR, respiratory rate (RR), SaO_2_, body weight, or body temperature have been developed for the rapid management of HF and COPD exacerbations. Frequently, the goal is to incorporate such models into a telemedicine platform.

The models developed to date can be defined as predictive (models predicting the medium-long term risk of exacerbations, when the patient is in a stable state) or diagnostic (models detecting an exacerbation episode which is already on course). Regarding diagnostic exacerbation models in HF [7,8,9,10,11] and COPD [12,13,14,15,16,17,18,19,20,21], they have shown variable and poor Sn ranging between 40–75%. Ledwidge et al. [22] developed a HF diagnostic exacerbation model with higher Sn (82%) though low Sp (68%). Similarly, Shah et al. [23] have developed a COPD model with 80% Sn but, again, low Sp (36%). The low Sn and Sp of these models restrict their use in routine clinical practice.

To improve the performance of such algorithms, the study of other clinical parameters has been proposed [12,24]. In this regard, no studies or algorithms developed up to now have considered the diagnostic performance of vital signs in effort situations (for example, while the patient is walking). Physical effort produces a physiological response (variations in the HR, RR or SaO_2_), which may be different depending on whether the patient is in a stable or an exacerbation phase of disease [25,26,27]. Additionally, physical effort may evidence alterations in certain parameters, which are not observed when the patient is evaluated at rest in the initial phases of an exacerbation episode. Therefore, the goal of the present study was to evaluate the differences in the physiological response to effort between patients in a stable/exacerbation phase of disease (HF and/or COPD) and, on the basis of such possible differences, to develop a model for detecting exacerbation episodes.

## 2. Experimental Section

### 2.1. Design and Sample

This study was designed as a prospective longitudinal trial. Subjects were recruited among patients who were hospitalized at the Internal Medicine, Cardiology or Pneumology Units with a main diagnosis of HF and/or COPD exacerbation (in this study, exacerbation was defined as a decompensation episode severe enough as to cause hospitalization). Included patients were older than 55 years and were able to walk for at least 30 m at the moment of evaluation. Patients with HF New York Heart Association (NYHA) functional class IV, patients with a pacemaker or intra-cardiac device, and patients on long-term oxygen therapy prior to recruitment were excluded.

Two third-level hospitals (university hospitals of reference at the national level with 300–1500 hospital beds and highly specialized units) plus two second-level hospitals (regional hospitals with 200–800 hospital beds, with 5 to 10 medical specialties including Internal Medicine, Cardiology and Pneumology) participated in the study, each one with a reference physician and a trained interviewer. Interviewers were in charge of visiting the corresponding center every day and contacting the reference physician in order to identify patients with a main diagnosis of HF or COPD exacerbation from the list of hospitalization units. Subsequently, the reference physician confirmed the diagnosis with the physicians in charge of those patients. Patients without such medical confirmation of the diagnosis were not included in the study.

We conducted a convenience sampling including all consecutive patients, who met the inclusion criteria, admitted to any of the participating hospitals during an 18-month period starting on November 2010. Assuming a case (patient in an exacerbation phase)/control (patient in a stable phase) proportion of 1, expected Sn and Sp of 80% and 90% respectively, and 10% accuracy with a 95% confidence level, a total sample size of 124 subjects was calculated.

### 2.2. Participants’ Evaluation and Variables

**Figure 1:**
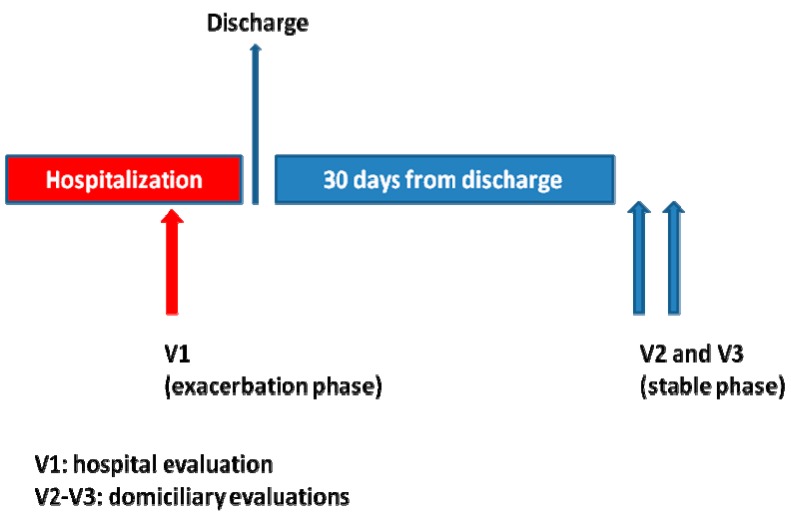
Timeline for evaluation sessions during the trial.

Every patient underwent three identical evaluation sessions in the following order (Figure 1): one session during hospital stay (V1), considered as an evaluation in an exacerbation phase, and two sessions at home (V2 and V3), considered as evaluations in a stable phase. V1 was carried out after the corresponding physician authorized the patient to walk (namely, after the most severe part of the process had passed). Thirty days after discharge, one of the research physicians contacted the participant via telephone to check whether he/she was in a stable phase. To this end, a questionnaire was administered, asking the patients whether, compared to the moment of hospital discharge, they had experienced increased cough, sputum, or dyspnea; increased the dose or re-started a corticoid therapy, initiated an antibiotic treatment, or visited the doctor due to the worsening of any clinical condition. If the answer to any of these questions was “yes”, it was considered that the patient was not in a stable state. In such case, sessions (V2, V3) were postponed until the researcher considered, based on successive telephone calls, that the patient was in a stable state. Sessions V2 and V3 were scheduled with at least 24 h between them.

The interviewers evaluated the patients within 24–72 h following medical confirmation of the diagnosis of exacerbation or stable phase.

Interviewers were trained by the researchers, through theoretical and practical sessions, on the process of data collection and the way of conducting the evaluation sessions with the patients. On one day during the first three months of recruiting, every interviewer was accompanied by a researcher, to verify that the training had been adequate. Throughout the study, a telephone line was available to interviewers, to contact the reference physician and one of the researchers.

Every evaluation session consisted of 3 consecutive phases: rest (patient sitting for at least 20 min), effort (walking on a flat non-tilted surface at the patient’s usual pace for a maximum period of 6 min) and recovery (4 min immediately following the termination of the effort phase, with the patient in the sitting position).

The main parameters that were evaluated were: RR, HR, SaO_2_, and walking distance (m). The interviewer measured SaO_2_ and HR by placing a pulse oximeter with memory function (Model 3100, Nonin Medical, Inc., Plymouth, MN, USA) on the patient’s left index finger at the end of the rest phase, just before the participant started walking. Both SaO_2_ and HR were measured continuously and per-minute means were subsequently calculated, using the device’s software, throughout the effort and recovery phases. Interviewers measured the RR at the end of every phase, while using a distraction maneuver (i.e., they pretended to palpate the radial pulse). Interviewers measured the total walking distance with an odometer, while walking alongside the patients. No specific walking route was established; patients were allowed to walk at their convenience, along the hospital corridor or at home. However, walking was stopped in cases where the patient showed HR higher than 220 beats/min, SaO_2_ lower than 85%, or dyspnea/pain that prevented them from continuing to walk. In cases where the patient was using oxygen therapy at the moment of evaluation, it was discontinued at least 15 min before they started walking.

Dyspnea was measured by using the NYHA scale [28], the modified Medical Research Council (mMRC) scale [29] and the Borg scale [30]. Blood pressure at rest, body weight, and body temperature were also recorded.

### 2.3. Statistical Analysis

Statistical analysis was carried out by splitting the sample into two groups, according to medical diagnosis (HF exacerbation/COPD exacerbation). Patients with both diagnoses were included in both groups.

Two types of discriminant analysis were conducted for every parameter. First, mean values from exacerbation (V1) and stable (V2) phases were compared (Student’s *T*-test for paired samples). Second, differences (V1–V2) and (V3–V2) were calculated, where the first ones were considered cases (i.e., the differences between exacerbation phase V1 and stable phase V2) and the latter ones were considered controls (i.e., the differences between stable phase V3 and stable phase V2). On the basis of the calculated differences, all possible cutpoints were evaluated and Sn (case group) and Sp (control group) were calculated for every cutpoint. Given that the prevalence of cases in the study (stable/exacerbation proportion 1:1) was clearly higher than that usually reported in clinical practice, predictive values were not analyzed.

From the second analysis, parameters with relevant discriminatory power were selected and their combined diagnostic performance was evaluated using a serial and parallel testing strategy. In this study, the serial testing strategy consists of first determining the absence (negative result) or presence (positive result) of a certain parameter and, only in case of a positive result, the absence or presence of a second parameter is determined. The final result is only considered to be positive in those cases in which both parameters are positive (in this way, net Sp is strengthened, although net Sn is lowered). In the parallel testing strategy, the presence (positive result) or absence (negative result) of two or more parameters is simultaneously determined, and it is enough that one of them is positive to consider the final result as positive (net Sn is strengthened although net Sp is lowered) [31]. The requisites for a parameter to be considered relevant were: Youden index [32] equal to or higher than 0.2 plus one of the following: Sn of at least 80% (for the parallel testing strategy) or Sp of at least 80% (serial testing strategy).

In order to control for the effects of bradycardia-inducing drugs (beta-blockers, verapamil, diltiazem, digoxin, and amiodarone) and assuming that baseline HR is elevated during the course of a cardiac/respiratory exacerbation, participants who had been on treatment with a bradycardia-inducing drug during the stable phase (V2, V3) but not the exacerbation phase (V1) were excluded from the HR analysis. Pain and anxiety were evaluated using the Face Pain Scale [33,34] and asking a direct question about the occurrence of anxiety. Outliers of physiological parameters RR (<8 breaths/min or >40 breaths/min) and rest HR (<30 beats/min or >150 beats/min), as well as lacking or lost results were excluded from the analysis.

Statistical analysis was conducted with the statistical software package SPSS Statistics 24 (IBM, Armonk, NY, USA).

This study was conducted in accordance with the amended Declaration of Helsinki and was approved by the Ethics Committee for Clinical Research of the Hospital of Mataró (Comité Ético de Investigación Clínica del Hospital de Mataró) (approval number: 1851806) and those of the other participating centers. Informed consent was obtained from all participants.

## 3. Results

### 3.1. Demographics

**Figure 2:**
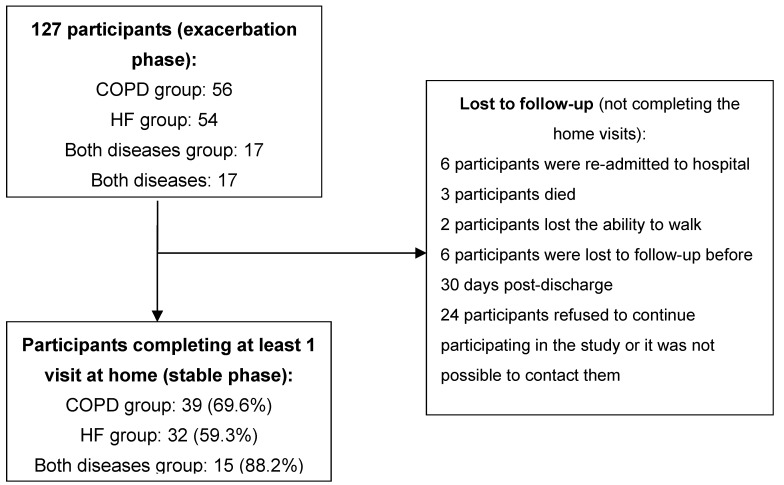
Participant recruiting and follow-up.

A total of 127 participants were recruited (56 with COPD exacerbation, 54 with HF exacerbation and 17 with exacerbation of both conditions). Figure 2 shows the number of participants who completed the follow-up and the reasons behind cases lost to follow-up. Table 1 shows the baseline results per HF or COPD condition.

### 3.2. Mean and Diagnostic Performance of Individual Parameters

**Figure 3:**
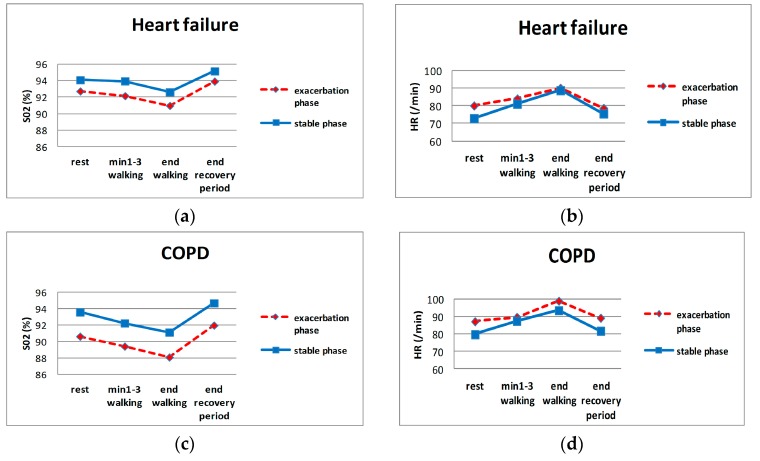
HR and SaO_2_ during evaluation sessions. (**a**,**b**): SaO_2_ and HR of HF patients, respectively; (**c**,**d**): SaO_2_ and HR of COPD patients, respectively.

Table 2 and Figure 3 show the means of the evaluated clinical parameters. All SaO_2_-related parameters varied significantly between the exacerbation vs. stable phase for both diseases, although the largest differences were for COPD.

Table 3 shows the clinical parameters with relevant discriminatory power. There were at least one SaO_2_, one HR, and one walking distance parameter considered as such, for both conditions. None of the parameters showed Sn and Sp higher than 80% simultaneously. Dyspnea, blood pressure, RR, and body weight did not show relevant discriminatory power. Changes between rest (patient seated) vs. effort (initiation of walking) and between effort (termination of walking) vs. recovery (patient resting seated) did not show relevant discriminatory power either, for differentiating the exacerbation from the stable phase.

It was not necessary to exclude any value due to outlier criteria; 14 patients were excluded from the HR analysis because they were on a treatment with a bradycardia-inducing drug in the stable phase but not in the exacerbation phase (8, 3 and 3 patients from the HF, COPD and both conditions groups, respectively). The frequency of pain and anxiety did not change significantly between the stable/exacerbation phases.

### 3.3. Diagnostic Performance of the Developed Algorithms

For both conditions, a diagnostic algorithm could be developed by following the parallel testing strategy. We studied up to 23 HF models and 16 COPD models. Table 4 shows HF and COPD models with the highest diagnostic performance and clinical consistency, according to the researchers’ opinion. The AUC of the COPD model was 0.921 (95% CI: 0.847–0.995) and that of the HF model was 0.841 (95% CI: 0.741–0.941).

It was not possible to develop a model with the serial testing strategy (only one parameter with Sn >80% and Youden index >0.2 was found).

## 4. Discussion

### 4.1. Main Results

An important observation about the discriminatory power of the here-presented models is that all parameters have to be evaluated in effort (HR, SaO_2_, and walking distance); this reinforces the proposal that physical exercise may have unmasking effects on potential HR and/or SaO_2_ alterations, which would not be observed at rest at the beginning of an exacerbation episode. Not differentiating between rest and effort could partially account for the failure to observe significant variations in these parameters during exacerbation, reported by other authors [12,24,35].

Parameter SaO_2_ shows the largest differences between the stable and exacerbation phases, especially in COPD. This finding is relevant because SaO_2_ can be easily measured. Although SaO_2_ drops during exacerbation episodes are well known, this study defines their discriminative power in quantitative terms of diagnostic performance, and, finding that discriminative power is important, they have been considered for the models. It is related with previous studies on HF and COPD. Masip et al. [9] demonstrated the importance of monitoring SaO_2_ to detect acute HF in intensive care units, and SaO_2_ has been described as the physiological variable with the highest discriminatory power in COPD [12]. Given that several of the physiological factors that determine SaO_2_ are affected in exacerbations of both conditions, it is not unexpected that monitoring SaO_2_ is useful in both of them.

Interestingly, the walking distance showed a noticeable discriminatory power. Although it has been shown that walking distance varies depending on whether the patient is in an exacerbation or a stable phase of both HF or COPD [36,37], this variable (6-min walking test [6 MWT]) has only been evaluated as a prognostic factor for mortality or readmission to hospital in both conditions [38,39,40,41] but not as a diagnostic parameter to detect an exacerbation episode which is already in course. The findings in this study describe this latter use, defining important realistic cutpoints (35 and 40 m for COPD and HF, respectively) considering the concept of minimal clinically important difference previously described in other studies (15–30 m [42]).

### 4.2. Clinical Usefulness/Feasibility

We conducted the present study bearing in mind the possible implementation of the developed models into a telemedicine system. In this regard, we consider that the Sn and Sp reported for the COPD model are relevant; although the HF ones are not, since the 75% Sp would result in a number of false-positive detections and consequent alarms, which hinder its clinical applicability. In terms of feasibility, the model could be implemented through a computer application on a mobile device (mobile phone), which would receive and process data from a pulse-oximeter (HR and SaO_2_). The walking distance could also be measured through the patient’s mobile phone.

### 4.3. Validity/Bias

Since the sample included patients admitted to second- and third-level hospitals, a wide range of the clinical spectrum of these conditions is expected to be represented. However, the models developed in this research were focused on patients with severe exacerbation episodes (those for which hospitalization is required); therefore, these models do not cover the population affected by milder exacerbations (for which ambulatory treatment is usually enough). Participants affected by exacerbations requiring hospitalization were selected because telemedicine systems are usually considered for patients with more severe disease. Additionally, since all participants included in this research already suffered from known HF or COPD, the models cannot be considered for people without HF or COPD background in their medical record (e.g., patients with new-onset HF or COPD who present an exacerbation episode).

We recognize the possibility of influencing the discriminatory power of the models by including participants with exacerbation of both conditions. However, because of the convergence of physiopathological mechanisms, we consider that the influence of this selection bias is limited. For example, in a study by Masip et al. [9] the SaO_2_ cutpoint for detecting HF did not change in patients that also suffered from COPD. Moreover, cases of patients simultaneously affected by both conditions are increasingly frequent.

Although guidelines for 6 MWT [43] recommend conducting two tests (learning effect has been demonstrated), only one test was allowed at hospital (exacerbation phase) because it was expected that participants would be too tired to repeat the walking session. Such limitation could adversely influence the discriminatory power of walking distance. However, this influence is expected to be minor since guidelines also recognize that when walking distance is used to assess hospitalization risk or mortality the learning effect may be less important and one test may be sufficient.

Additionally, as the reference for the stable phase, we used the patient’s situation after an exacerbation episode, where a potential latent functional deterioration might influence the walking distance, as compared with the distance that would be measured before exacerbation. However, such a bias would make walking distance an even more sensitive discriminatory parameter.

### 4.4. Limitations

Due to the restricted sample size, we used all the data to develop the algorithms; thus, their validation is still pending. Evaluations in the exacerbation phase were conducted after the most severe part of an episode had passed, because that was similar to the beginning of an exacerbation episode, which is the most important moment to take action. We recruited participants in an exacerbation phase and we did not start with a cohort in a stable phase because of the cost and effort associated with the follow-up (not all patients would suffer exacerbation during follow-up or some could take a long time to do so). Considering this design, a specific timeframe for the model (e.g., one week before hospitalization occurred) could not be defined.

## 5. Conclusions

Monitoring physiological variables during effort situations (e.g., walking) may improve the discriminatory power of algorithms based on such physiological parameters for the detection of exacerbation episodes of both HF and COPD.

SaO_2_ shows the largest differences between the stable and exacerbation phases, especially in COPD. Discriminatory power in quantitative terms of diagnostic performance has been defined.

The diagnostic models presented here should be considered preliminary; they need further study and timeframes for them to be defined.

## Figures and Tables

**Table 1:** Distribution of baseline characteristics according to disease.

	HF (*n* = 54)	COPD (*n* = 56)	Both (*n* = 17)	*p*
Age (SD)	78.4 (8.3)	73.4(8.4)	75.8(9.6)	0.010
Male (%)	24 (44.4)	43 (76.8)	16 (94.1)	<0.01
Diabetes mellitus 2 (%)	27 (50.0)	11 (19.6)	6 (35.3)	0.002
Dyslipidemia (%)	16 (29.6)	21 (37.5)	5 (29.4)	0.707
Active smoking (%)	2 (3.7)	13 (23.2)	1 (5.9)	0.008
Treatment with bradycardia-inducing drug (previous to current exacerbation) (%)	37 (68.5)	7 (12.5)	12 (70.6)	<0.01
Previous hospitalization due to cardiac/respiratory disease (SD)	0.9 (1.2)	1.9 (1.9)	1.7 (1.4)	0.007
Length of stay in days (SD)	9 (8.1)	11.3 (11.4)	7.9 (7.0)	0.445
Baseline evaluation time (days previous to discharge) (SD)	2.3 (3.8)	3.8 (4.4)	2.9 (2.7)	0.299
Dyspnea (NYHA) (%)				
I	16 (29.6)	16 (28.6)	5 (29.4)	0.924
II	22 (40.7)	20 (35.7)	8 (47.1)	
III	16 (29.6)	20 (35.7)	4 (23.5)	
Dyspnea (mMRC) (%)				
0	12 (22.2)	14 (25)	4 (23.5)	0.391
1	6 (11.1)	9 (16.1)	2 (11.8)	
2	13 (24.1)	16 (28.6)	4 (23.5)	
3	23 (42.6)	17 (30.4)	7 (41.2)	
Body mass index (SD)	26.7 (4.6)	25.2 (4.4)	26.8 (8.2)	0.235
Osteoarthritis (%)	35 (64.8)	25 (44.6)	7 (41.2)	0.055

Dyspnea results correspond to the degree of limitation at the moment of baseline evaluation (exacerbation phase at hospital). HF: heart failure; COPD: chronic obstructive pulmonary disease; NYHA: New York Heart Association; mMRC: modified Medical Research Council; SD: standard deviation; %: percentage; *n*: sample size.

**Table 2:** Physiological parameters according to disease and exacerbation/stable phase.

Clinical Parameter	HF				COPD			
	Exacerb. Phase (SD)	Stable Phase (SD)	Mean Difference (95% CI)	*p*	Exacerb. phase (SD)	Stable Phase (SD)	Mean Difference (95% CI)	*p*
Oxygen saturation (%)								
Rest	92.7 (3.4)	94.1 (3.2)	−1.36 (−2.42; −0.3)	0.01	90.6 (3.2)	93.6 (2.6)	−2.96 (−3.77; −2.16)	<0.001
Minute 1 to 3 of effort (mean)	92.2 (3.6)	94 (3.1)	−1.79 (−2.65; −0.93)	<0.001	89.4 (3.9)	92.2 (3.2)	−2.84 (−3.91; −1.77)	<0.001
End-of-effort	91 (5.6)	92.6 (4.1)	−1.64 (−2.82; −0.46)	0.01	88.1 (5.1)	91.1 (4.3)	−3.04 (−4.16; −1.91)	<0.001
Mean-of-effort period	91.2 (3.7)	92.8 (3.4)	−1.63 (−2.45; −0.81)	<0.001	88.7 (3.7)	91.3 (3.5)	−2.57 (−3.42; −1.72)	<0.001
End-of-recovery	93.9 (2.9)	95.1 (2.5)	−1.23 (−1.96; −0.51)	<0.001	92 (3.1)	94.7 (2.5)	−2.72 (−3.56; −1.87)	<0.001
Mean of recovery period	92.3 (3.4)	93.7 (3.2)	−1.42 (−2.28; −0.57)	<0.001	90.5 (3.5)	93 (3.1)	−2.48 (−3.39; −1.57)	<0.001
Heart rate (beats/min) ^(1)^								
Rest	80.1 (12.2)	72.9 (11.4)	7.22 (2.11; 12.33)	0.01	87.3 (15.4)	79.9 (14.5)	7.4 (3.05; 11.74)	<0.001
Minute 1 to 3 of effort (mean)	84.1 (9.9)	81.2 (13.9)	2.95 (−2.6; 8.49)	0.29	89.8 (14.6)	87.5 (16.8)	2.21 (−3.45; 7.88)	0.43
End-of-effort	90.2 (12.3)	88.9 (16.1)	1.28 (−4.91; 7.47)	0.68	99 (16.2)	93.6 (16.8)	5.42 (0.84; 9.99)	0.02
Mean-of-effort period	87.2 (9.5)	83.2 (13.1)	3.97 (−0.74; 8.68)	0.1	93.3 (12.4)	89.8 (14.7)	3.47 (−1.18; 8.11)	0.14
End-of-recovery	78.7 (14.6)	75.6 (13.3)	3.06 (−2.13; 8.24)	0.24	89.3 (14.9)	81.9 (13.2)	7.4 (3.05; 11.76)	<0.001
Mean of recovery period	83.5 (11.6)	80 (13.9)	3.5 (−2.01; 9.01)	0.21	89.4 (13.6)	86.2 (14.4)	3.19 (−1.42; 7.8)	0.17
Respiratory rate (breaths/min)								
Rest	21.4 (5.1)	20.8 (4.5)	0.57 (−0.74; 1.88)	0.38	21.2 (5.1)	21.2 (4.6)	0.02 (−0.92; 0.95)	0.97
End-of-effort	24.5 (5.3)	23.6 (5.3)	0.85 (−0.43; 2.13)	0.19	24.7 (5.6)	23.9 (5)	0.85 (−0.07; 1.77)	0.07
End-of-recovery	22 (5.5)	21.3 (4.7)	0.65 (−0.78; 2.08)	0.36	22.5 (4.6)	21.3 (4.1)	1.19 (0.21; 2.17)	0.02
Distance (m)								
Walking distance	126.7 (89.8)	133.1 (82.5)	−6.36 (−31.3; 18.57)	0.61	134.1 (93.8)	157.9 (100.2)	−23.78 (−49.99; 2.42)	0.07
Dyspnea								
NYHA scale	2.1 (0.9)	1.9 (0.7)	0.17 (−0.05; 0.39)	0.13	2.1 (0.9)	1.8 (0.7)	0.28 (0.01; 0.55)	0.04
mMRC scale	2 (1.2)	1.7 (1.1)	0.28 (−0.02; 0.57)	0.06	1.7 (1.4)	1.5 (1.3)	0.24 (−0.15; 0.63)	0.22
Borg scale (rest)	1.7 (1.3)	1.7 (1.5)	−0.09 (−0.61; 0.44)	0.75	1.6 (1.3)	1.6 (1.3)	0.04 (−0.45; 0.52)	0.88
Borg scale (effort)	2.7 (1.9)	3.1 (2.4)	−0.43 (−1.2; 0.35)	0.27	3.3 (2.5)	2.9 (2.4)	0.41 (−0.45; 1.27)	0.35
Blood pressure								
Systolic pressure	120.1 (19.6)	125.1 (23.6)	−5.04 (−10.75; 0.66)	0.08	125.4 (18.3)	126.7 (20.3)	−1.28 (−6.85; 4.29)	0.65
Diastolic pressure	70.4 (13.3)	70 (13.4)	0.38 (−3.77; 4.53)	0.85	73.8 (10.8)	73.7 (12.1)	0.07 (−3.33; 3.48)	0.97

For cases in which the difference between means reached significance, the differences between both stable-phase visits (V2 vs. V3) were calculated in order to determine whether the observed differences corresponded to biological variability. No case differences due to biological variability were found except for the end-of-recovery respiratory rate, both in COPD and HF patients. (1): Heart rate values from patients, who were indicated treatment with a bradycardia-inducing drug upon discharge but not during hospital stay, were excluded (14 patients). HF: heart failure, COPD: chronic obstructive pulmonary disease, NYHA: New York Heart Association, mMRC: modified Medical Research Council, SD: standard deviation; CI: confidence interval.

**Table 3:** Parameters with relevant discriminatory power ^(1)^ for detection of clinical exacerbation.

Clinical Parameter	Cutpoint ^(2)^	HF				COPD			
		*n*	Sn (95% CI)	*n*	Sp (95% CI)	*n*	Sn (95% CI)	*n*	Sp (95% CI)
**Oxygen saturation (%)**									
Rest	≥1					54	0.85 (0.73–0.92)	46	0.72 (0.57–0.83)
	2	47	0.53 (0.39–0.67)	43	0.86 (0.73–0.93)				
	3	47	0.34 (0.22–0.48)	43	0.95 (0.85–0.99)				
Minute 1 to 3 of effort (mean)	2	35	0.4 (0.26–0.56)	33	0.91 (0.76–0.97)	35	0.57 (0.41–0.72)	29	0.93 (0.78–0.98)
End-of-effort	3					54	0.5 (0.37–0.63)	46	0.87 (0.74–0.94)
Mean-of-effort period	2	46	0.46 (0.32–0.6)	40	0.88 (0.74–0.95)				
End-of-recovery	2	47	0.51 (0.37–0.65)	43	0.88 (0.76–0.95)				
	3	47	0.28 (0.17–0.42)	43	0.95 (0.85–0.99)	53	0.53 (0.4–0.66)	46	0.89 (0.77–0.95)
Mean of recovery period	2	45	0.47 (0.33–0.61)	40	0.88 (0.74–0.95)				
	3	45	0.31 (0.2–0.46)	40	0.93 (0.8–0.97)	48	0.44 (0.31–0.58)	38	0.89 (0.76–0.96)
**Heart rate (beats/min)**									
Rest	≥10	36	0.42 (0.27–0.58)	32	0.84 (0.68–0.93)				
	15	36	0.36 (0.22–0.52)	32	0.91 (0.76–0.97)	48	0.31 (0.2–0.45)	41	0.95 (0.84–0.99)
Minute 1 to 3 of effort (mean)	10	31	0.42 (0.26–0.59)	26	0.92 (0.76–0.98)	33	0.3 (0.17–0.47)	27	0.93 (0.77–0.98)
End-of-effort	10					48	0.4 (0.27–0.54)	41	0.93 (0.81–0.97)
Mean-of-effort period	10	35	0.4 (0.26–0.56)	29	0.97 (0.83–0.99)				
Mean of recovery period	10	34	0.35 (0.21–0.52)	29	0.93 (0.78–0.98)				
	15	34	0.26 (0.15–0.43)	29	1 (0.88–1)				
**Walking distance (m)**									
Walking distance difference	≥35	44	0.34 (0.22–0.49)	42	0.95 (0.84–0.99)	51	0.43 (0.31–0.57)	46	0.93 (0.82–0.98)
	40	44	0.34 (0.22–0.49)	42	0.98 (0.88–1)				

(1): Sn or Sp ≥ 80%, and Youden index ≥ 0.20. (2): cutpoints refer to oxygen saturation decrease of at least 1, 2 or 3 points as compared to the stable phase, heart rate increase of at least 10–15 beats/min as compared to the stable phase and walking distance decrease of at least 35–40 m as compared to the stable phase. HF: heart failure, COPD: chronic obstructive pulmonary disease, Sn: sensitivity, Sp: specificity, CI: confidence interval; *n*: sample size.

**Table 4:** Diagnostic performance of the developed clinical algorithms.

Disease	Clinical Parameter	Cutpoint	*n*	Sn (95% CI)	*n*	Sp (95% CI)	ROC Area (95% CI)
HF	Oxygen saturation decrease (mean-of-effort period) of at least 2 points;	at least 1 condition	33	0.85 (0.69–0.93)	28	0.75 (0.57–0.87)	0.841 (0.741–0.941)
	heart rate increase (mean-of-effort period) of at least 10 beats/min;						
	Walking distance decrease of at least 40 m						
COPD	oxygen saturation decrease (average of minute 1 to 3 of effort period) of at least 2 points	at least 1 condition	31	0.9 (0.75–0.97)	27	0.89 (0.72–0.96)	0.921 (0.847–0.995)
	heart rate increase (end-of-effort) of at least 10 beats/min						
	Walking distance decrease of at least 35 m						

HF: heart failure, COPD: chronic obstructive pulmonary disease; Sn: sensitivity; Sp: specificity; CI: confidence interval; ROC: Receiver Operating Characteristic; *n*: sample size.
